# Omega 3 Consumption and Anxiety Disorders: A Cross-Sectional Analysis of the Brazilian Longitudinal Study of Adult Health (ELSA-Brasil)

**DOI:** 10.3390/nu10060663

**Published:** 2018-05-24

**Authors:** Lara Natacci, Dirce M. Marchioni, Alessandra C. Goulart, Maria Angélica Nunes, Arlinda B. Moreno, Letícia O. Cardoso, Luana Giatti, Maria del Carmen B. Molina, Itamar S. Santos, André R. Brunoni, Paulo A. Lotufo, Isabela M. Bensenor

**Affiliations:** 1 Postgraduate student, School of Medicine, University of São Paulo (USP), São Paulo SP 05508-000, Brazil; lara@dietnet.com.br; 2 Department of Nutrition, School of Public health, University of São Paulo (USP), São Paulo SP 01246-904, Brazil; marchioni@usp.br; 3 Center for Clinical and Epidemiological Research, University Hospital, University of São Paulo (USP), São Paulo SP 05508-000, Brazil; alecgoulart@yahoo.com.br (A.C.G.); Brazil; itamarss@gmail.com (I.S.S.); palotufo@usp.br (P.A.L.); 4 Postgraduate Program in Epidemiology, School of Medicine, Federal University of Rio Grande do Sul (UFRS), Porto Alegre RS 90035-003, Brazil; maanunes@gmail.com; 5 Department of Epidemiology and Quantitative Methods in Health, National School of Public Health Sérgio Arouca, Fundação Oswaldo Cruz, Rio de Janeiro RJ 90035-003, Brazil; morenoar@uol.com.br (A.B.M.); leticiaocar@ensp.fiocruz.br (L.O.C.); 6 Postgraduate Program in Public Health; General Hospital, School of Medicine, Federal University of Minas Gerais (UFMG), Belo Horizonte MG 30130-100, Brazil; luana.giatti@gmail.com; 7 Departament of Nutrition, Federal University of Espírito Santo (UFES), Vitória ES 29043-900, Brazil; mdmolina@uol.com.br; 8 Laboratory of Neurosciences (LIM-27), Department and Institute of Psychiatry, University of São Paulo (USP), São Paulo SP 05403-010, Brazil; brunowsky@gmail.com

**Keywords:** anxiety disorders, mental disorders, polyunsaturated fatty acids, ω-3 fatty acids, nutrition intake

## Abstract

Few studies have evaluated the association between diet and mental disorders, and it has been established that ω-3 (*n*-3) fatty acids may have a beneficial effect for sufferers of anxiety disorders. This study is part of the Brazilian Longitudinal Study of Adult Health (ELSA-Brasil)—a population-based cohort study on diet and mental health—and searched for associations between anxiety disorders and consumption of *n*-3 polyunsaturated fatty acids (PUFA). The study had a cross-sectional design, with a total sample of 12,268 adults. Dietary exposure was measured by a quantitative food-frequency questionnaire, and mental diagnoses were assessed by the Clinical Interview Schedule—Revised Version and diagnosed according to the International Classification of Diseases (ICD-10). Logistic regression models were built using quintiles of *n*-3, ω 6 (*n*-6), *n*-6/*n*-3 ratio, and PUFA, using the 1st quintile as reference. Anxiety disorders were identified in 15.4% of the sample. After adjusting for sociodemographic variables, cardiovascular risk factors, diet variables, and depression, intakes in the 5th quintile were inversely associated with anxiety disorders for EPA (OR = 0.82, 95% CI = 0.69–0.98), DHA (OR = 0.83, 95% CI = 0.69–0.98), and DPA (OR = 0.82, 95% CI = 0.69–0.98). Participants in the fifth quintile of *n*-6/*n*-3 ratio had a positive association with anxiety disorders. Although results suggest a possible protective effect of *n*-3 fatty acids against anxiety, all associations lost significance after adjustment for multiple comparisons.

## 1. Introduction

Diet has been studied as an important risk factor for cardiovascular disease and diabetes [1], but more recently, studies have been conducted to analyze the association between diet and mental health.

Few studies have evaluated the association between diet and anxiety disorders [2,3,4]. Some recent analyses have focused on the consumption of ω-3 (*n*-3) fatty acids such as the α-linolenic acid (ALA), eicosapentaenoic acid (EPA), docosapentaenoic (DPA), and docosahexaenoic acid (DHA), and their association with anxiety disorders [5,6]. A Spanish cohort study with 7903 participants conducted a prospective analysis of the relationship between long-chain *n*-3 fatty acids and fish intake with the incidence of mental disorders after a two-year follow-up [5]. Results may suggest a beneficial effect of moderate intake of *n*-3 in mental disorders, including anxiety, which was not confirmed in the final models. Recently, Jacka et al. reported an association between high consumption of docosahexaenoic acid (DHA) and less anxiety in a cross-sectional analysis between intake of fish, *n*-3 and omega 6 (*n*-6) fatty acids, and anxiety disorders in Australian women, and reported an inverse association between DHA intake and anxiety disorders [6]. In a randomized clinical trial, consumption of 3 g of *n*-3 fatty acids, mainly EPA and DHA, was associated with significant decreases in anger and anxiety scores compared to placebo [7]. However, another clinical trial showed no association between EPA intake and anxiety disorders in the intervention compared to the placebo group [8]. In a double blind, randomized, controlled, 8-week study, Lespérance et al. did not find benefits of omega-3 supplementation in patients experiencing both major depressive disorders and anxiety disorders [9].

The plausibility of the association among *n*-3 PUFA intake and protection against psychiatric disorders may be related to their possible effect in the modulation of the communication of brain neurons [10]. *n*-3 PUFAs play a critical role in brain function and structure throughout a person’s lifetime by regulating neurotransmission, neurogenesis, cell survival, and neuroinflammation [11]. In relation to anxiety, they may be particularly important in regulating stress responses. Studies have shown that the acute stress response is mitigated by fish oil supplementation [12]. Studies using animal models have also showed that diets with poor *n*-3 fatty acids may be associated with changes in rat behavior due to an alteration in the endocannabinoid system [13]. Other studies in rats suggested that diets rich in *n*-6 PUFA are associated with depressive and anxious behaviors in rats [14], compared to a protective effect for these behaviors in diets rich in *n*-3 fatty acids [15].

A systematic review and meta-analysis of the epidemiology of mental disorders that examined 174 studies from 1980–2013 in 63 different countries, led by Steel et al. [16], found that on average, one in five adults had suffered mental disorders in the previous 12 months, and 29.2% suffered from mental disorders across their lifetime. In Brazil, anxiety disorders are especially common, representing the eighth highest cause of disability-adjusted life-years, which is higher than in most other countries [17].

The Brazilian Longitudinal Study of Adult Health (ELSA-Brasil) is a cohort study that provides information about diet and mental health in 15,105 civil servants in the baseline examination. It is an interesting setting for a cross-section evaluation of the association between *n*-3 and *n*-6 fatty acids, and fish intake with anxiety disorders at baseline. We hypothesized that high consumption of *n*-3 polyunsaturated fatty acids (PUFA) may be associated with lower levels of anxiety disorders.

## 2. Materials and Methods

The ELSA-Brasil is designed to investigate the incidence of cardiovascular diseases, diabetes, and their biological and social determinants. Originally, 15,105 subjects aged 35–74 years from six cities—Belo Horizonte, Porto Alegre, Rio de Janeiro, Salvador, São Paulo, and Vitória—were included in the study [18,19,20,21]. In this analysis, we included cross-sectional data from the baseline examination of the study, which was conducted from August 2008 to December 2010.

Sample size estimation was based on the main study outcomes—type 2 diabetes and myocardial infarction. We conservatively estimated a 3-year cumulative incidence of 1.4%. Considering an alpha value of 5%, statistical power of 80%, exposure prevalence of 20%, and a relative risk of 2.0, we estimated the necessary sample size at approximately 6400 subjects. To present gender-specific analyses and allow for possible losses to follow-up, we defined the desired sample size as approximately 15,000 persons. This study was conducted according to the guidelines laid down in the Declaration of Helsinki and all procedures involving human subjects/patients were approved by the “Comitê de Ética em Pesquisa” (Institutional Review Board) under the number 659/06. Written or verbal informed consent was obtained from all subjects/patients. Where verbal consent was obtained, it was witnessed and formally recorded.

### 2.1. Diet Measurement

Dietary exposure was measured by a quantitative food-frequency questionnaire (FFQ), which included 114 food items relating to consumption in the past 12 months [22]. The reproducibility and relative validity of The Food Frequency Questionnaire (FFQ) used in the Brazilian Longitudinal Study of Adult Health (ELSA-Brazil) was evaluated in a study with 281 participants. Reproducibility and validity were assessed by the intra-class correlation coefficient (ICC) and the FFQ showed satisfactory reliability for all nutrients and reasonable validity, especially for energy, macronutrients, calcium, potassium, and vitamins E and C [23]. To determine nutrient intake based on the FFQ, the number of servings consumed per day × weight (standard portion in grams) × frequency of consumption × nutritional composition of the food serving was calculated. The daily equivalent coefficients used were: 3 for more than 3 times/day, 2.5 for 2 to 3 times/day, 1 for once/day, 0.8 for 5 to 6 times/week, 0.4 for 2 to 4 times/week, 0.1 for once/week, 0.07 for 1 to 3 times/month, and 0 for never/almost never [22]. 

The Food Frequency Questionnaire (FFQ) of ELSA-Brazil used the Nutrition Data System for Research (NDSR) software (University of Minnesota, Minneapolis, USA, 2010). The adjustment for energy intake was done using the residual method [24].

Using data from the FFQ, we quantified the daily intake of fish and seafood (g/day), *n*-6 (linoleic acid-g/day and arachidonic acid-g/day), *n*-3 fatty acids including α-linolenic-g/day, eicosapentaenoic (EPA-g/day), docosapentaenoic (DPA-g/day), and docosahexaenoic (DHA-g/day), as well as all polyunsaturated fatty acids. We also calculated the *n*-6/*n*-3 ratio.

### 2.2. Healthy Eating Index

The revised version of the Brazilian Healthy Eating Index (BHEI) is an indicator of dietary quality that was developed according to current nutritional recommendations. The Revised Index consists of twelve components: nine food groups included in the 2006 Brazilian Dietary Guidelines, in which daily portions are expressed in terms of energy density; two nutrients (sodium and saturated fats); and calories from solid fat, alcohol, and added sugar. The Revised Brazilian Healthy Eating Index allows for the measurement of dietary risk factors for chronic diseases by evaluating and monitoring the diet at both individual and population levels [25].

The following participants were excluded from the analysis: those using *n*-3 or *n*-6 fatty acid supplements, those who reported previous bariatric surgery, those who consumed less than 500 or more than 4000 calories per day [26], and those for whom information on the main variables used in the study was missing.

### 2.3. Anxiety and Depression Diagnosis

Mental diagnoses were assessed by lay interviewers trained by a psychiatrist with experience in epidemiological studies, using the validated, Portuguese version of the Clinical Interview Schedule Revised (CIS-R) [27]. The CIS-R is a structured questionnaire for measuring and diagnosing non-psychotic psychiatric morbidity in the community. It is a short, straightforward questionnaire that was developed by Lewis et al. [28] specifically for use in community and primary care settings. Importantly, lay interviewers are as reliable as psychiatrists in using the CIS-R to perform mental diagnoses, making it a suitable instrument for use in our cohort. The complete CIS-R version was applied, allowing us to diagnose mental disorders corresponding to the following categories of the tenth version of the International Classification of Diseases (ICD-10) [29]: major depressive disorder (MDD, F32); general anxiety disorder (GAD, F41.1); panic disorder (F41.0); social anxiety disorder (F40.1); phobias (F40.2); and obsessive-compulsive disorder (F42). In our analysis, participants were compared in relation to the absence or presence of any anxiety disorder. An anxiety disorder was considered to be present if the participant had one of the following: general anxiety disorder, panic disorder, social anxiety disorder, phobia, or obsessive-compulsive disorder.

### 2.4. Other Variables

A comprehensive set of questionnaires, tests, and measurements [21] were carried out. The questionnaire included information on age at the baseline visit (years), self-reported race/skin color (White, Mixed race, Black, Asian, and Indigenous), educational achievement (less than high-school, high school and some college, or at least completed college), smoking status (never, past, or current), alcohol intake (never, past, or current), and physical activity, which was measured using the leisure time section of the long version of the International Physical Activity Questionnaire [30]. 

Weight and height were measured with the participant wearing light clothes. Body mass index (BMI) was calculated by dividing weight in kilograms by height in meters squared (kg/m^2^). Blood pressure (BP) was measured using a validated Omron HEM 705CPINT oscillometer device. Three measurements were taken at one-minute intervals, and the mean of the two latter blood pressure measurements was considered as the value for defining hypertension. Hypertension was defined as use of medication to treat hypertension or systolic blood pressure ≥ 140 mm Hg, or diastolic blood pressure ≥ 90 mm Hg. Diabetes was defined as a previous medical history of diabetes, use of medication to treat diabetes, fasting plasma glucose ≥ 126 mg/dL, 2-h plasma glucose ≥ 200 mg/dL, or HbA_1C_ ≥ 6.5%. Dyslipidaemia was defined as LDL cholesterol ≥130 or use of medication to treat dyslipidaemia [31].

### 2.5. Laboratory Measurements

After overnight fasting (8–14 h), plasma samples were collected from all the participants. Fasting glucose was determined enzymatically by the hexokinase method. Total cholesterol, high-density lipoprotein cholesterol (HDL-cholesterol), and triglycerides were determined using the enzymatic colorimetric method. Low-density lipoprotein cholesterol (LDL-C) was calculated using the Friedewald equation, or directly when triglyceride levels were greater than 400 g/dL. High sensitivity CRP was determined using immunochemistry (nephelometry) [31].

### 2.6. Statistical Analysis

The categorical variables were expressed as frequencies and percentages, and compared using the chi-square test. The continuous variables were presented as a mean (standard deviation) or median (interquartile range), and compared using ANOVA with the post hoc test of Bonferroni for parametric variables and Kruskal-Wallis test for non-parametric variables, after assessing normality assumptions. Triglycerides were log-transformed for comparison, but the values in the tables have been back-transformed to their natural scales. Diet variables measured in g/day were adjusted for total energy intake [24] and then categorized in quintiles.

Logistic regression models were built using quintiles of *n*-3 and *n*-6, and fish and seafood consumption, as well as all polyunsaturated fatty acids, and the *n*-6/*n*-3 ratio was found using the first quintile as a reference. Model 1 consists of the Odds Ratio (OR) and 95% Confidence Intervals (95% CI), which were expressed as crude values, adjusted by age, sex, race, and education. Model 2 consists of all the variables in model 1, plus cardiovascular risk factors, such as smoking, alcohol use, physical activity, hypertension, diabetes, dyslipidaemia, BMI, and total energy. Lastly, the fully adjusted model, Model 3, included all variables in Model 2 with further adjustment for quality of diet and major depressive disorder. We performed a sensitive analysis using continuous data for nutrients in logistic models to verify linear trends. We used a Holm-Bonferroni test to adjust results for multiple comparisons. The analyses were carried out using the software SPSS 20.0 (IBM, Chicago, IL, USA). *p* < 0.05 was considered significant, and all the tests were two-sided.

## 3. Results

Of the 15,105 participants initially included in the study, we excluded 139 participants who were using supplements with *n*-3 or *n*-6, 107 who reported previous bariatric surgery, 33 who reported consumption of less than 500 calories/day, 1414 who reported consumption of more than 4000 calories/day, 28 with missing information in the food-frequency questionnaire, 170 with missing information about anxiety disorders, 64 with missing information about intake of *n*-3 or *n*-6 supplements, and 24 with missing information about bariatric surgery. As some of the participants satisfied more than one exclusion criteria, the total number of participants included in the analysis was 12,268, of which 1893 (15.4%) had anxiety disorders and 10,375 (84.6%) did not.

Table 1 shows the general characteristics of the participants included in the analysis. Most of the participants with anxiety disorders were women (74.1%). Participants with anxiety reported a lower frequency of ≥11 years of education, and of being white. They also practiced less vigorous physical activity, had a higher frequency of current smoking, and had a lower frequency of current alcohol consumption compared to participants without anxiety. Participants with anxiety also presented slightly higher BMI and CRP-values compared to those without anxiety. The distribution of cardiovascular risk factors was similar in both groups. Median values of EPA, DPA, DHA, and the *n*-6/*n*-3 ratio are slightly lower in the participants with anxiety compared to those without anxiety (Table 2).

When we used the continuous variables for nutrients (g), we detected a linear trend for *n*-3, arachidonic acid, EPA, DPA, DHA, and *n*-6/*n*-3 ratio (*p* < 0.05 for all). No linear trend was detected for PUFA, linoleic acid, alfa-linolenic acid, and *n*-6 (*p* > 0.05 for all of them).

Table 3 shows the odds ratio (OR) and 95% confidence intervals (95% CI) for the association of anxiety disorders with intake of *n*-3 and *n*-6 fatty acids, presented as crude values and after multivariate adjustment for possible confounders. After multivariate adjustment for sociodemographic variables (age, sex, race, and education), lifestyle (body-mass index, smoking, alcohol intake, and physical activity), total calories, and quality of the diet, the participants in the fifth quintile of EPA and DHA intake, and in the fourth and fifth quintiles of DPA, showed an inverse relationship between anxiety disorders and intake of *n*-3: OR 0.81 (95% CI, 0.68–0.95), OR 0.80 (95% CI, 0.68–0.95), and OR 0.81 (95% CI, 0.69–0.95), respectively. Participants in the fifth quintile of *n*-6/*n*-3 ratio showed a direct association with anxiety disorders OR 1.23 (95% CI, 1.04–1.45). After further adjustment for depression, the results remained similar for EPA OR 0.82 (95% CI, 0.69–0.98), DPA OR 0.83 (95% CI, 0.69–0.98), DHA OR 0.82 (95% CI, 0.69–0.98), and *n*-6/*n*-3 ratio OR 1.21 (95% CI, 1.01–1.44). For DPA, even in the fourth quintile, there was an inverse relationship with anxiety disorders in model 2 and model 3 with OR 0.80 (95% CI, 0.68–0.94) and OR 0.82 (95% CI, 0.69–0.97), respectively.

After multivariate adjustment, all associations lost significance: linoleic (*p* = 0.99), alpha-linolenic (*p* = 0.99), arachidonic (*p* = 0.54), EPA (*p* = 0.32), DPA (*p* = 0.86), DHA (*p* = 0.27), *n*-3 (*p* = 0.55), *n*-6 (*p* = 0.99), *n*-6/*n*-3 ratio (*p* = 0.32) and PUFA (*p* = 0.99).

## 4. Discussion

Our study shows an inverse association between intake of *n*-3, EPA, DPA, and DHA and anxiety disorders after multivariate adjustment for sociodemographic variables, cardiovascular risk factors, total energy, quality of diet, and depression. We also found an inverse association between higher *n*-6/*n*-3 ratio and the presence of anxiety disorders, which corroborate our results for EPA, DPA, and DHA, even after adjustment for depression. However, after further adjustments for multiple comparisons, all associations in our results lost significance.

Few studies have evaluated the relationship between anxiety disorders and consumption of *n*-3 and *n*-6 polyunsaturated fatty acids, with conflicting results [5,6,7,8]. Compared to our study, some of these studies have used screening questionnaires that did not permit anxiety disorders to be “ruled in” according to the Diagnostic and Statistical Manual of Mental Disorders or the ICD-10. Our results are similar to a cross-sectional analysis of Jacka et al. in Australian women, that also reported an inverse association between DHA intake and anxiety disorders, although each study used different scales to evaluate anxiety disorders. Jacka et al. [6] reported an association between high DHA intake and fewer anxiety disorders, while in our results, a higher intake of EPA, DPA, and DHA was associated with fewer anxiety disorders. Although DHA represents the most abundant *n*-3 polyunsaturated fatty acid in the human brain [32], the only trial with *n*-3 supplementation for anxiety disorders to show positive results used both DHA and EPA as part of the supplementation (corroborating our results in relation to EPA) [7]. However, our results lost significance after adjustment for multiple comparisons, being similar to a Spanish study that investigated prospectively the association between *n*-3 intake and mental disorders, finding some positive results that were not confirmed in final models [5]. In the study by Sanchez-Villegas [5], they used a simple question about a self-reported previous medical diagnosis of depression, anxiety, stress, or use of antidepressants and tranquilizers at baseline and after a 2-year follow-up.

Most evidence of the association between *n*-3 fatty acids and psychiatric disorders was provided by studies analyzing this association in depressive disorders. In a recent meta-analysis of observational studies, Grosso et al. support the hypothesis that dietary *n*-3 PUFA intake is associated with lower risk of depression [33]. However, Appleton et al., in a meta-analysis including only clinical trials, concluded that, at present, we do not have sufficient high-quality evidence to determine the effects of *n*-3 PUFA as a treatment for major depressive disorders [34]. Although studies analyzing the association of *n*-3 fatty acids with depressive disorders are more frequent than studies analyzing the association with anxiety disorders, results are still conflicting.

These conflicting results are still present in the evidence provided by other types of study. Reduced erythrocyte *n*-3 PUFA concentrations of α-linolenic, EPA, and DHA have been associated with the severity of social anxiety disorder measured on the Liebowitz Social Anxiety Scale in 27 patients compared to 22 patients without anxiety. Patients with depressive disorders were excluded from the sample [35]. Liu et al. [36] identified in adults with major depressive disorder (MDD), with and without comorbid DSM-IV anxiety disorders and healthy controls, that the presence and severity of comorbid anxiety were negatively associated with EPA and DHA levels. No association was found with depression. Therefore, there are still conflicting data between the association of *n*-3 fatty acids and depression and anxiety disorders using different types of studies [5,6,7,37]. Most of these studies include small samples and are cross-sectional.

Our study has some limitations. It is a cross-sectional analysis that only permits the study of association, but not of causality. Information about diet was measured at one single point in time. FFQs are prone to recall bias since individuals are asked to report their intake retrospectively and usually refer to prolonged periods of time. Additionally, FFQs can be hampered by the individual’s intentional misreporting of their consumption of certain foods and could result in a differential misclassification with unpredicted consequences in the estimated associations. However, our study also has some strengths. We used a questionnaire for mental disorders that permits psychiatric diagnosis according to the ICD-10 in a large sample of men and women. In our study, 15.4% of the population had at least one anxiety disorder, like other studies in Brazil, which enhanced our power to describe significant associations [38,39]. We were able to exclude participants using *n*-3 and *n*-6 supplements from the analyses. We also adjusted the logistic regression models for a comprehensive set of confounders, including quality of diet, and beyond that, for the presence of a major depressive disorder and a further adjustment for multiple comparisons. Generalizability of our data is more probable since ELSA-Brasil includes a sample of apparently healthy volunteers compared to analysis in more specific samples.

## 5. Conclusions

In this cross-sectional analysis, higher intake of *n*-3 polyunsaturated fatty acids EPA, DHA, and DPA were inversely associated with anxiety disorders. The higher *n*-6/*n*-3 ratio was directly associated with the presence of these disorders. Although results suggest a possible protective effect of *n*-3 fatty acids against anxiety, all associations lost significance after adjustment for multiple comparisons. Future prospective data in ELSA-Brasil may help to clarify a possible protective effect of *n*-3 PUFA against anxiety disorders.

## Figures and Tables

**Table 1:** Clinical characteristics of participants according to the presence or absence of anxiety disorders. ELSA-Brazil, 2018.

Characteristics	Anxiety Disorders		*p*-Value
	Yes	No	
	*N* = 1893	*N* = 10,375	
Age (years) *	51.0 (8.5)	52.5 (9.2)	<0.0001
Women (%)	1402 (74.1)	5761 (55.5)	<0.0001
Self-reported race (%)			<0.0001
White	913 (48.6)	5714 (55.7)	
Mixed	561 (29.9)	2719 (26.5)	
Black	332 (17.7)	1450 (14.1)	
Asian	40 (2.1)	288 (2.8)	
Native	31 (1.7)	86 (0.8)	<0.0001
Education (%)			
Less than high school	251 (13.3)	1147 (11.1)	
High school and some college	762 (40.3)	3172 (30.6)	
At least complete college	880 (46.5)	6056 (58.4)	
Hypertension (%)	684 (36.1)	3653 (35.2)	0.44
Diabetes (%)	385 (20.3)	1995 (19.2)	0.26
Dyslipidaemia (%)	1073 (57.1)	6072 (59.0)	0.14
Smoking (%)			<0.0001
Never	1027 (54.3)	6049 (58.3)	
Past Current	558 (29.5)	3102 (29.9)	
Current	308 (16.3)	1223 (11.8)	
Alcohol intake (%)			<0.0001
Never	234 (12.4)	1094 (10.5)	
Past	464 (24.5)	1943 (18.7)	
Current	1194 (63.1)	7336 (70.7)	
Physical activity at leisure (%)			<0.0001
Mild	1535 (82.4)	7738 (75.7)	
Moderate	203 (10.9)	1518 (14.9)	
Vigorous	125 (6.7)	961 (9.4)	
Body Mass Index(kg/m^2^) *	27.4 (5.0)	26.9 (4.6)	<0.0001
Systolic blood pressure (mm Hg)	119.3 (17.0)	120.1 (17.1)	<0.0001
Diastolic blood pressure (mm Hg)	75.6 (10.7)	75.8 (10.5)	0.37
Fasting plasma glucose mg/dL (16.1)/dL	110.9 (31.9)	111.7 (30.2)	0.32
LDL-cholesterol * mg/dL	130.8 (33.8)	131.2 (34.8)	0.60
HDL-cholesterol * mg/dL	57.5 (14.0)	57.2 (14.7)	0.36
Triglycerides mg/dL ^£^	110 (74–170)	113 (75–180)	0.06

Notes: Hypertension: Use of medications to treat hypertension or systolic blood pressure ≥ 140 mm Hg or diastolic blood pressure ≥ 90 mm Hg. Diabetes: Previous medical history of diabetes or use of medication to treat diabetes, fasting blood glucose ≥ 126 mg/dL, postprandial blood glucose of ≥200 mg/dL, or HbA_1C_ ≥ 6.5%. Dyslipidaemia: LDL cholesterol ≥ 130 or use of medication to treat dyslipidaemia. ^£^ median and interquartile range; * mean and standard deviation. LDL-cholesterol: Low-intensity lipoprotein; HDL-cholesterol: High-intensity lipoprotein [29].

**Table 2:** Median and Interquartile range of intake of fish in terms of *n*-3, *n*-6, *n*-6/*n*-3 ratio, monounsaturated and polyunsaturated fatty acids, according to the presence or absence of anxiety. ELSA-Brazil, 2018.

Characteristics	Anxiety Disorders		*p*-Value
	Yes	No	
	*N* = 1893	*N* = 10375	
Linoleic acid g/day	15.51 (12.89–18.39)	15.44 (12.77–18.39)	0.46
Arachidonic acid g/day	0.26 (0.17–0.38)	0.26 (0.17–0.41)	0.01
α-linolenic acid g/day	2.30 (1.94–2.74)	2.31 (1.95–2.72)	0.34
EPA g/day	0.13 (0.05–0.21)	0.14 (0.06–0.27)	<0.0001
DPA	0.12 (0.05–0.21)	0.13 (0.06–0.23)	<0.0001
DHA g/day	0.42 (0.14–0.79)	0.46 (0.20–0.88)	<0.0001
PUFA g/day	19.13 (16.17–22.43)	19.2 (16.17–22.61)	0.48
*n*-3 total	3.01 (2.42–3.84)	3.10 (2.47–4.03)	<0.0001
*n*-6 total	15.77 (13.14–18.66)	15.72 (13.05–18.73)	0.57
*n*-6/*n*-3 ratio	5.19 (4.15–6.45)	5.10 (3.97–6.27)	<0.0001

Note: Continuous variables were presented as a median (interquartile range) and compared using ANOVA with the post-hoc Mann-Whitney test.

**Table 3:** Logistic regression, showing the odds ratio (95% confidence interval) of the association between anxiety disorder and linoleic acid, arachidonic acid, alpha-linolenic acid, EPA, DPA, DHA, PUFA, *n*-3, *n*-6 consumption, and *n*-6/*n*-3 ratio (ELSA-Brasil, 2018).

	Quantity (g)	Crude	Model 1	Model 2	Model 3
Linoleic acid					
1^st^ quintile	4.36–12.79	1.0 (Reference)	1.0 (Reference)	1.0 (Reference)	1.0 (Reference)
2^nd^ quintile	12.80–14.62	1.07 (0.91–1.25)	1.07 (0.91–1.26)	1.06 (0.90–1.25)	1.13 (0.95–1.34)
3^rd^ quintile	14.63–16.29	1.08 (0.93–1.27)	1.08 (0.92–1.26)	1.05 (0.89–1.23)	1.07 (0.90–1.28)
4^th^ quintile	16.30–18.39	1.10 (0.94–1.28)	1.12 (0.96–1.32)	1.08 (0.92–1.27)	1.09 (0.91–1.29)
5^th^ quintile	18.40–37.94	1.06 (0.90–1.24)	1.10 (0.94–1.30)	1.05 (0.89–1.25)	1.07 (0.89–1.27)
Arachidonic acid					
1^st^ quintile	0.00–0.17	1.0 (Reference)	1.0 (Reference)	1.0 (Reference)	1.0 (Reference)
2^nd^ quintile	0.18–0.23	0.95 (0.81–1.10)	0.95 (0.82–1.12)	0.93 (0.79–1.09)	0.95 (0.80–1.12)
3^rd^ quintile	0.24–0.30	0.97 (0.83–1.13)	1.00 (0.85–1.16)	0.98 (0.84–1.15)	1.01 (0.85–1.19)
4^th^ quintile	0.31–0.40	0.96 (0.82–1.12)	0.97 (0.83–1.14)	0.96 (0.81–1.12)	0.99 (0.84–1.17)
5^th^ quintile	0.41–1.89	**0.79 (0.68–0.93)**	**0.83 (0.71–0.98)**	**0.84 (0.71–0.99)**	0.85 (0.71–1.01)
Alpha-linolenic acid					
1^st^ quintile	0.95–1.95	1.0 (Reference)	1.0 (Reference)	1.0 (Reference)	1.0 (Reference)
2^nd^ quintile	1.96–2.19	0.96 (0.82–1.12)	0.96 (0.82–1.12)	0.98 (0.83–1.15)	0.99 (0.84–1.17)
3^rd^ quintile	2.20–2.42	1.01 (0.87–1.18)	1.01 (0.86–1.18)	1.02 (0.87–1.19)	1.01 (0.86–1.20)
4^th^ quintile	2.43–2.72	0.91 (0.78–1.06)	0.89 (0.76–1.05)	0.89 (0.75–1.05)	0.89 (0.75–1.06)
5^th^ quintile	2.73–5.49	0.92 (0.78–1.07)	0.92 (0.79–1.08)	0.93 (0.79–1.10)	0.95 (0.80–1.13)
EPA					
1^st^ quintile	0.00–0.06	1.0 (Reference)	1.0 (Reference)	1.0 (Reference)	1.0 (Reference)
2^nd^ quintile	0.07–0.11	0.93 (0.80–1.08)	0.95 (0.81–1.10)	0.94 (0.80–1.10)	0.96 (0.81–1.13)
3^rd^ quintile	0.12–0.17	0.94 (0.80–1.09)	0.98 (0.84–1.14)	0.99 (0.85–1.16)	1.04 (0.88–1.23)
4^th^ quintile	0.18–0.27	**0.84 (0.72–0.97)**	0.90 (0.77–1.05)	0.93 (0.79–1.09)	0.93 (0.78–1.10)
5^th^ quintile	0.28–2.27	**0.71 (0.61–0.83)**	**0.77 (0.66–0.91)**	**0.81 (0.68–0.95)**	**0.82 (0.69–0.98)**
DPA					
1^st^ quintile	0.00–0.06	1.0 (Reference)	1.0 (Reference)	1.0 (Reference)	1.0 (Reference)
2^nd^ quintile	0.07–0.10	0.92 (0.79–1.06)	0.93 (0.80–1.08)	0.93 (0.80–1.09)	0.97 (0.82–1.15)
3^rd^ quintile	0.11–0.15	0.91 (0.78–1.06)	0.94 (0.81–1.09)	0.95 (0.81–1.11)	0.96 (0.82–1.14)
4^th^ quintile	0.16–0.23	**0.74 (0.64–0.87)**	**0.78 (0.67–0.91)**	**0.80 (0.68–0.94)**	**0.82 (0.69–0.97)**
5^th^ quintile	0.24–1.84	**0.72 (0.61–0.84)**	**0.77 (0.65–0.90)**	**0.80 (0.68–0.95)**	**0.83 (0.69–0.98)**
DHA					
1^st^ quintile	0.00–0.20	1.0 (Reference)	1.0 (Reference)	1.0 (Reference)	1.0 (Reference)
2^nd^ quintile	0.21–0.37	0.89 (0.76–1.03)	0.91 (0.78–1.06)	0.91 (0.78–1.06)	0.94 (0.80–1.11)
3^rd^ quintile	0.38–0.54	0.95 (0.82–1.10)	0.99 (0.85–1.15)	1.01 (0.86–1.18)	1.02 (0.87–1.20)
4^th^ quintile	0.55–0.86	**0.77 (0.66–0.90)**	**0.83 (0.71–0.97)**	0.85 (0.72–1.00)	0.87 (0.73–1.03)
5^th^ quintile	0.87–7.13	**0.71 (0.61–0.83)**	**0.77 (0.66–0.91)**	**0.81 (0.69–0.95)**	**0.82 (0.69–0.98)**
PUFA					
1^st^ quintile	7.08–16.17	1.0 (Reference)	1.0 (Reference)	1.0 (Reference)	1.0 (Reference)
2^nd^ quintile	16.18–18.25	1.03 (0.88–1.20)	1.03 (0.88–1.21)	1.02 (0.87–1.20)	1.08 (0.91–1.28)
3^rd^ quintile	18.26–20.14	1.01 (0.86–1.18)	1.01 (0.86–1.18)	0.99 (0.84–1.16)	1.00 (0.84–1.18)
4^th^ quintile	20.15–22.29	1.03 (0.88–1.20)	1.08 (0.92–1.26)	1.04 (0.88–1.22)	1.03 (0.87–1.22)
5^th^ quintile	22.60–46.13	0.94 (0.80–1.10)	0.99 (0.84–1.17)	0.96 (0.81–1.13)	0.96 (0.81–1.43)
*n*-6/*n*-3 ratio					
1^st^ quintile	0.54–4.00	1.0 (Reference)	1.0 (Reference)	1.0 (Reference)	1.0 (Reference)
2^nd^ quintile	4.01–4.80	1.12 (0.95–1.31)	1.10 (0.93–1.29)	1.89 (0.92–1.29)	1.10 (0.92–1.31)
3^rd^ quintile	4.81–5.45	1.24 (1.06–1.46)	1.20 (1.02–1.42)	1 17 (0.99–1.38)	1.18 (0.99–1.41)
4^th^ quintile	5.46–6.29	1.20 (1.03–1.41)	1.16 (0.98–1.36)	1.11 (0.94–1.31)	1.13 (0.94–1.34)
5^th^ quintile	6.30–13.98	**1.33 (1.14–1.56)**	**1.29 (1.10–1.52)**	**1.23 (1.04–1.45)**	**1.21 (1.01–1.44)**
*n*-3					
1^st^ quintile	1.06–2.46	1.0 (Reference)	1.0 (Reference)	1.0 (Reference)	1.0 (Reference)
2^nd^ quintile	2.47–2.88	0.98 (0.84–1.14)	1.02 (0.88–1.19)	1.02 (0.87–1.19)	1.06 (0.90–1.25)
3^rd^ quintile	2.89–3.32	0.94 (0.81–1.09)	0.98 (0.84–1.14)	1.00 (0.86–1.17)	1.01 (0.86–1.19)
4^th^ quintile	3.33–4.00	0.88 (0.75–1.03)	0.94 (0.80–1.10)	0.94 (0.80–1.10)	0.96 (0.81–1.14)
5^th^ quintile	4.01–13.14	**0.75 (0.64–0.88)**	**0.82 (0.70–0.97)**	0.85 (0.72–1.00)	0.86 (0.72–1.02)
*n*-6					
1^st^ quintile	5.07–13.06	1.0 (Reference)	1.0 (Reference)	1.0 (Reference)	1.0 (Reference)
2^nd^ quintile	13.07–14.92	1.07 (0.91–1.25)	1.06 (0.90–1.24)	1.05 (0.89–1.24)	1.12 (0.95–1.33)
3^rd^ quintile	14.93–16.59	1.08 (0.92–1.26)	1.06 (0.91–1.25)	1.03 (0.88–1.22)	1.07 (0.90–1.27)
4^th^ quintile	16.60–18.72	1.09 (0.93–1.28)	1.12 (0.95–1.32)	1.08 (0.91–1.27)	1.10 (0.92–1.30)
5^th^ quintile	18.73–38.05	1.03 (0.88–1.21)	1.07 (0.91–1.26)	1.02 (0.87–1.21)	1.04 (0.87–1.24)

Note: Model 1: Adjustment for socio-demographic variables (age, gender, race and education). Model 2: Adjustment for sociodemographic variables, cardiovascular risk factors (hypertension, diabetes, dyslipidaemia, smoking, alcohol intake, and physical activity), and total calories. Model 3: Adjustment for sociodemographic variables, cardiovascular risk factors, calories, quality of diet, and depression.

## Founding

The ELSA-Brazil baseline study was supported by the Brazilian Ministry of Health (Science and Technology Department) and the Brazilian Ministry of Science and Technology (Studies and projects financier and CNPq National Research Council) (M.I.S., grant number 01 06 0010.00 RS; E.M.A., 01 06 0212.00 BA; J.G.M., 01 06 0300.00 ES; S.M.B., 01 06 0278.00 MG; D.C. 1 06 0115.00 SP, 01 06 0071.00 RJ). Brazilian Ministry of Health had no role in the design, analysis, or writing of this article. Brazilian Ministry of Science and Technology had no role in the design, analysis, or writing of this article. ARB receives a CAPES-Humboldt Research Fellowship for Experienced Researchers.
